# Regulation of viral replication by host restriction factors

**DOI:** 10.3389/fimmu.2025.1484119

**Published:** 2025-01-23

**Authors:** Ying Lin, Yun Zhu, Ling Jing, Xiaobo Lei, Zhengde Xie

**Affiliations:** ^1^ National Health Commission (NHC) Key Laboratory of System Biology of Pathogens and Christophe Merieux Laboratory, National Institute of Pathogen Biology, Chinese Academy of Medical Sciences and Peking Union Medical College, Beijing, China; ^2^ Key Laboratory of Major Diseases in Children, Ministry of Education, National Clinical Research Center for Respiratory Diseases, Laboratory of Infection and Virology, Beijing Pediatric Research Institute, Beijing Children’s Hospital, Capital Medical University, National Center for Children’s Health, Beijing, China; ^3^ Research Unit of Critical Infection in Children, Chinese Academy of Medical Sciences, Beijing, China; ^4^ Key Laboratory of Pathogen Infection Prevention and Control (Peking Union Medical College), Ministry of Education, Beijing, China

**Keywords:** host restriction factors, antiviral, innate immune response, interferon, host-virus interaction

## Abstract

Viral infectious diseases, caused by numerous viruses including severe acute respiratory syndrome coronavirus 2 (SARS-CoV-2), influenza A virus (IAV), enterovirus (EV), human immunodeficiency virus (HIV), hepatitis B virus (HBV), and human papillomavirus (HPV), pose a continuous threat to global health. As obligate parasites, viruses rely on host cells to replicate, and host cells have developed numerous defense mechanisms to counteract viral infection. Host restriction factors (HRFs) are critical components of the early antiviral response. These cellular proteins inhibit viral replication and spread by impeding essential steps in the viral life cycle, such as viral entry, genome transcription and replication, protein translation, viral particle assembly, and release. This review summarizes the current understanding of how host restriction factors inhibit viral replication, with a primary focus on their diverse antiviral mechanisms against a range of viruses, including SARS-CoV-2, influenza A virus, enteroviruses, human immunodeficiency virus, hepatitis B virus, and human papillomavirus. In addition, we highlight the crucial role of these factors in shaping the host-virus interactions and discuss their potential as targets for antiviral drug development.

## 1 Introduction

Viruses, as obligate intracellular parasites, depend on host factors for their replication and survival. In turn, hosts have evolved various defensive strategies to control viral infection and spread, one of the most important is host restriction factors (HRFs) (1). HRFs are typically host proteins that limit viral replication. These HRFs are classified into two types: interferon-stimulated genes (ISGs) and non-interferon-stimulated genes (non-ISGs). To date, more than 1000 ISGs have been identified in mammals, many of which are restriction factors that specifically exhibit antiviral activity within infected cells (2). On the other hand, non-ISGs have also been found to inhibit viral infections (3). Unlike ISGs, these non-ISGs are constitutively expressed in cells and are not induced by interferons. Both ISGs and non-ISGs play essential roles in viral clearance, contributing to the host’s defense against viral infections. These factors interfere with various stages of the viral life cycle, including binding, entry, uncoating, transcription, translation, replication, assembly and release, ultimately inhibiting the replication and spread of viruses.

High-throughput screening methodologies, such as cDNA genome-wide gain-of-function screens, RNA interference, and CRISPR-Cas9 genome-wide loss-of-function screens, have significantly contributed to the discovery of numerous HRFs that impede the replication of various viruses such as HIV-1, IAV, CoV, and RSV (4–8). Notable HRFs include IFN-induced transmembrane proteins (IFITMs); surface-hinged, rigidly-extended killer proteins (SHREKs); IFN-induced proteins with tetratricopeptide repeats (IFITs); tripartite motif-containing proteins (TRIMs); and oligoadenylate synthetase (OAS) family proteins. Recent studies have shown that many HRFs effectively inhibit the replication of severe acute respiratory syndrome coronavirus 2 (SARS-CoV-2) infection (9). The discovery of these effective antiviral factors provides promising targets for broad-spectrum antiviral therapy. Understanding the molecular mechanisms of HRFs is crucial for developing host-targeting antiviral therapies (HATs). By targeting specific host-virus interactions, researchers have identified and continue to refine a range of viral antagonists with improved bioactivity and safety profiles (10). For example, a series of small molecule inhibitors have been proposed to inhibit HIV by stabilizing the expression of apolipoprotein B mRNA-editing enzyme catalytic polypeptide 3G (APOBEC3G, A3G) (11, 12). This review aims to highlight the recently elucidated antiviral mechanisms of these HRFs and discuss their implications for the development of novel antiviral drugs, thereby stimulating further research in this promising field.

## 2 HRFs that inhibit viral attachment or entry

**Figure 1:**
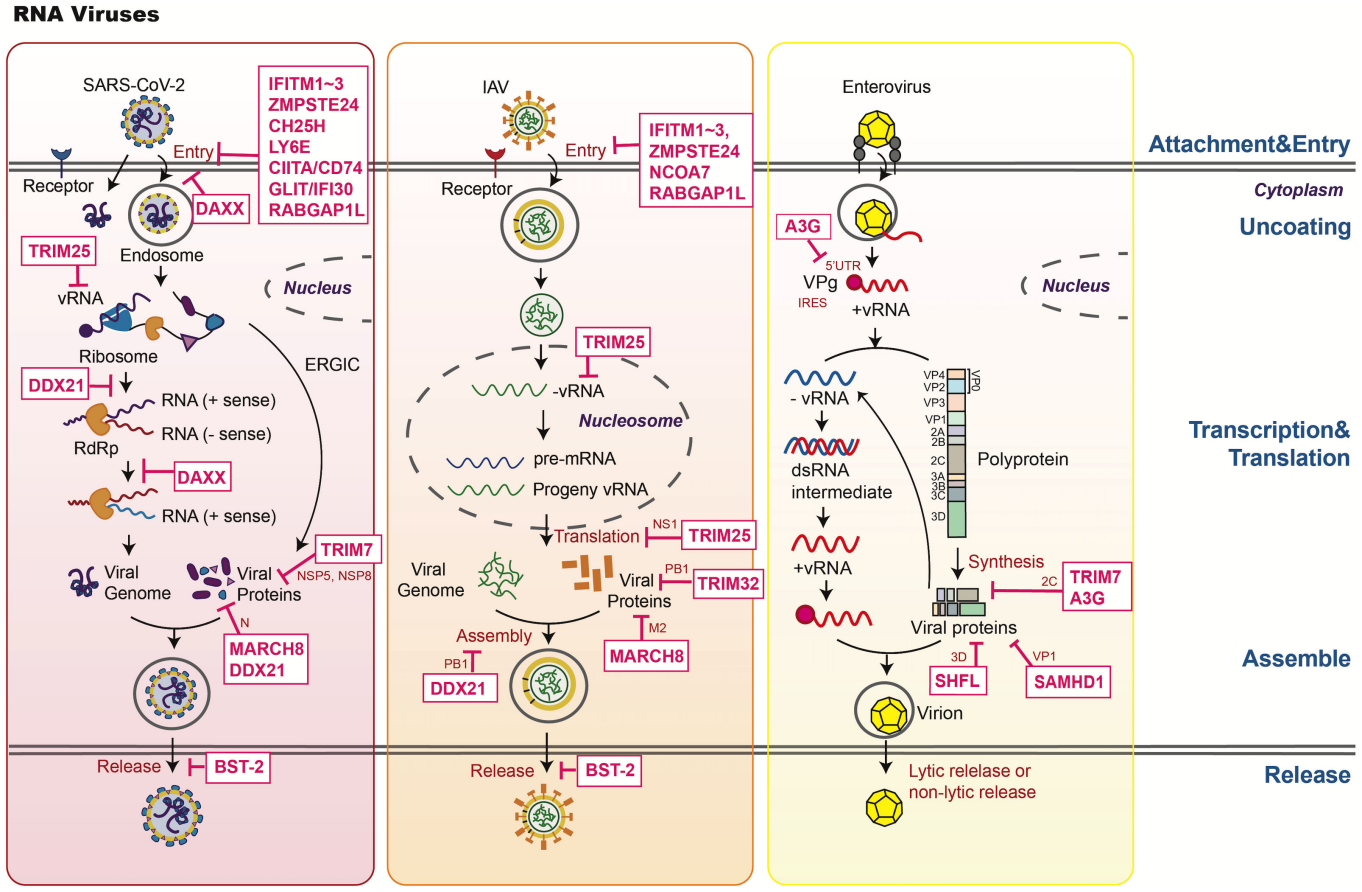
An overview of host restriction factors of RNA viruses. This figure illustrates the key mechanisms by which host restriction factors (HRFs) combat SARS-CoV-2, IAV, and enterovirus. HRFs inhibit viral replication through the following mechanisms: 1. Blocking attachment and entry (e.g., IFITMs, ZMPSTE24, CH25H, LY6E, DAXX). 2. Regulating replication and transcription. Inhibiting RNA synthesis through mechanisms like APOBEC3 deamination and TRIM7-mediated viral protein degradation. 3. Restricting assembly and release. Tethering viruses to the membrane (e.g., BST-2). 4. Modulating host signaling: Enhancing antiviral responses (e.g., TRIM25, TRIM14, TRIM7, DAXX).

**Figure 2:**
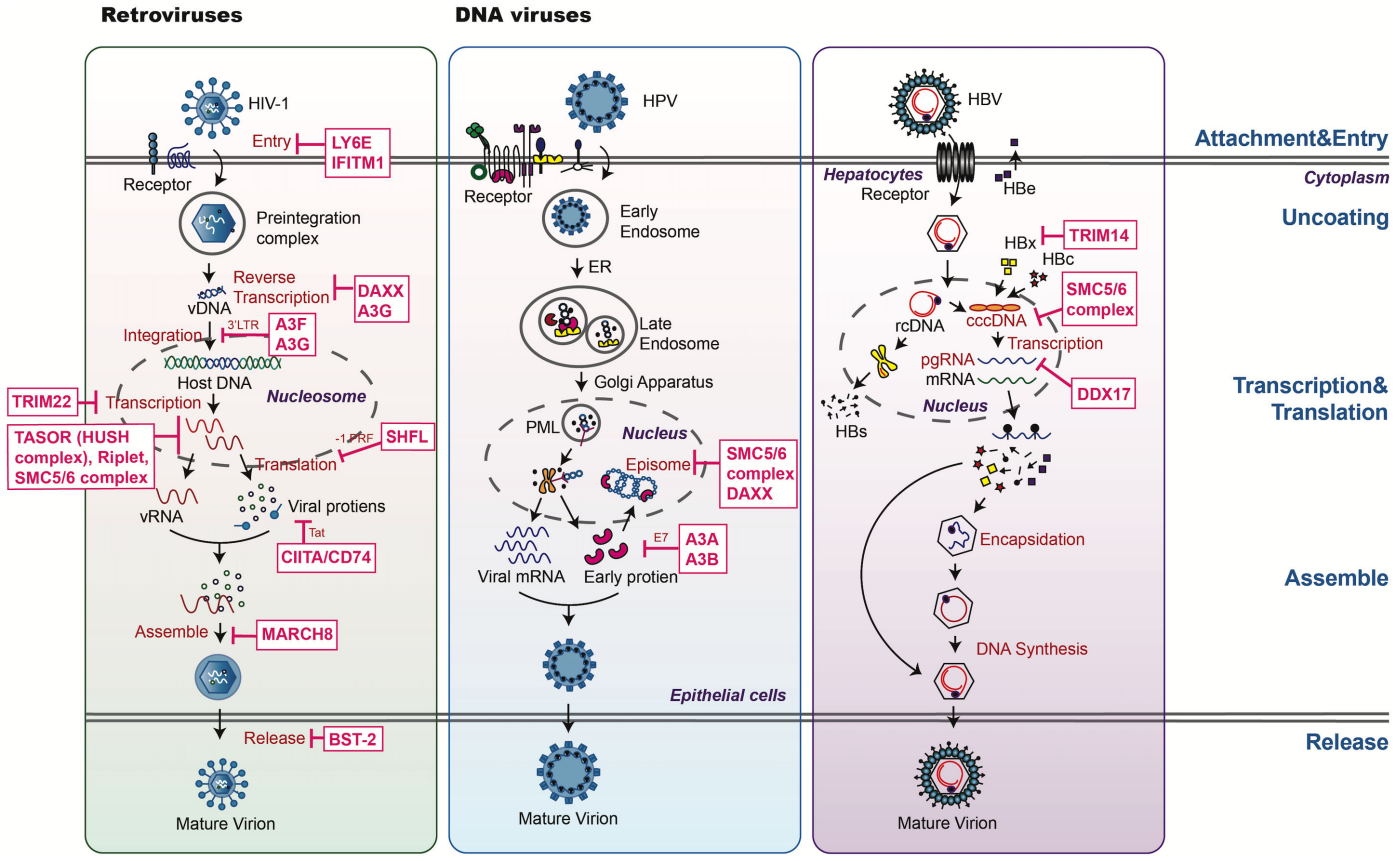
An overview of host restriction factors of retroviruses and DNA viruses. This figure illustrates key mechanisms by which host restriction factors (HRFs) combat HIV, HPV, and HBV. HRFs target various stages of the viral life cycle, including: 1. Blocking viral entry and nuclear import: preventing viral binding or fusion (e.g., IFITMs, LY6E). 2. Regulating replication and transcription: HRFs such as DAXX and the SMC5/6 complex suppress viral genome transcription through epigenetic silencing and chromatin remodeling. SAMHD1 reduces nucleotide pools required for viral DNA synthesis. 3. HRFs disrupt capsid assembly and prevent viral particle release (e.g., the SMC5/6 complex). 4. Modulating host defenses: proteins like OPTN mediate autophagy and ubiquitin-dependent degradation, targeting key viral components for destruction. The diagram categorizes HRFs by their antiviral roles, with dashed lines representing the targeting process.

Viral entry, comprising attachment and penetration, is the first step in establishing a successful infection. Non-enveloped viruses typically bind to specific receptors on the cell surface and enter through receptor-mediated clathrin- or dynamin-dependent endocytosis. In contrast, enveloped viruses fuse with the cell membrane before entering through internalization. Several HRFs specifically target this entry process. Examples include the IFITM family proteins, Zinc metallopeptidase STE24 (ZMPSTE24), Cholesterol 25-hydroxylase (CH25H), Lymphocyte antigen 6E (LY6E), Nuclear receptor coactivator protein 7 (NCOA7), Interferon-γ-inducible protein 30 (IFI30), and RAB GTPase-activating protein 1-like (RABGAP1L) (13). These HRFs prevent viral entry by preventing membrane fusion, inhibiting endosomal vesicle trafficking, impairing lysosomal function via reduced cathepsin activity, altering vesicle acidity, and directly modifying cell membrane dynamics (**Figures 1**, **2**). By disrupting these essential steps, HRFs effectively block the initial stages of viral infection, highlighting their importance in antiviral defense.

### 2.1 Interferon-induced transmembrane proteins

IFITMs, members of the dispanin/CD225 superfamily, include IFITM1, IFITM2, IFITM3, IFITM5, and IFITM10. Among these, IFITM1, IFITM2, and IFITM3 are notable for their roles as antiviral HRFs. First reported as interferon-stimulating genes (ISGs) in 1984, their roles as antiviral HRFs were identified through a siRNA screen against the influenza A(H1N1) virus in 2009 (14). IFITM1, IFITM2, and IFITM3 are highly homologous, sharing over 90% sequence identity; they function as broad-spectrum HRFs that restrict a wide range of viruses, including orthomyxoviruses, paramyxoviruses, rhabdoviruses, flaviviruses, filoviruses, poxviruses, and coronaviruses. Their primary mechanism of action involves blocking the fusion of viral envelopes with host cell membranes. This is achieved through an alpha-helix structure at the N-terminus, which functions as a wedge to alter membrane properties (e.g., rigidity and fluidity). A conserved GxxxG motif in these proteins is essential for their self-oligomerization, a critical process in their antiviral activity (15, 16). Compounds such as amphotericin B, which modulate membrane rigidity, can antagonize IFITM proteins. Additionally, IFITMs can inhibit viral biosynthesis of proteins and genes, or incorporate into virions to reduce the infectivity (16). IFIMTs can be activated by types I IFNs through an interferon-stimulated response element (ISRE) located in the 5’ promoter region of IFITM genes. This activation enhances the role of IFITMs in antiviral immunity. However, IFITMs are not able to restrict the replication of all viruses, such as Sendai virus (SeV), papillomavirus, cytomegalovirus, adenovirus, and arenavirus (13). The underlying mechanisms remain unclear and require further investigation to uncover the reasons behind their selective restriction capabilities.

IFITM1, IFITM2, and IFITM3 demonstrate varying antiviral potency against different viruses. For example, IFITM3 is more effective at inhibiting IAV and Zika virus (ZIKV), whereas IFITM1 exhibits stronger antiviral effects against HIV-1 and SARS-CoV-2 (17). Compared with IFITM2 and IFITM3, IFITM1 is more sensitive to the Alpha and Delta variants of SARS-CoV-2, although all three proteins can restrict the Omicron variant (18). The sensitivity of the Omicron variant to IFITMs is presumably determined by the S2 domain of its Spike protein (18). The antiviral activities of IFITM1, IFITM2, and IFITM3 are also influenced by their intracellular trafficking patterns. Palmitoylation, a crucial post-translational modification involving three cysteine residues, facilitates efficient binding of IFITM proteins to membrane lipids. This modification allows IFITM proteins to recognize membrane microdomains (e.g., lipid rafts) and target intracellular vesicles containing viruses, redirecting those vesicles to endolysosomes for degradation. IFITMs induced by IFN characterized with conserved cysteine residues that can be S-palmitoylated are necessary to manifest their anti-viral activities, like Cys72 of IFITM3 (19). Mutations in these cysteine residues compromise the stability of IFITM peptides, altering their subcellular localization and antiviral efficacy. Considering there are no compounds that target IFITMs, enzymes or compounds that regulate this reversible lipid modification process of IFITMs may be the potential broad-spectrum antiviral candidates.

### 2.2 Zinc metallopeptidase STE24

Zinc metallopeptidase STE24 (ZMPSTE24), also known as FACE1, is a constitutively expressed transmembrane protein predominantly localized to the nuclear and endosomal membranes. It is a zinc-dependent metalloprotease involved in critical cellular processes, including the biogenesis of lamin A, movement of misfolded proteins, and immune regulation. Recent evidence has shown that ZMPSTE24 defends cells against a broad spectrum of enveloped viruses, including vesicular stomatitis virus (VSV), flaviviruses such as ZIKV and Ebola virus (EBOV), vaccinia virus, IAV, SARS-CoV-2, and arenavirus, but not adenovirus or murine leukemia virus (20–22). The antiviral function of ZMPSTE24 is independent of its enzymatic activity and primarily relies on its cooperation with IFITM proteins. ZMPSTE24 acts as a crucial cofactor for IFITM1, IFITM2, and IFITM3, amplifying their antiviral effects by restricting viral entry through the restriction of the viral-membrane fusion process. Compared with IFITMs, ZMPSTE24 is more effective against VSV and less effective against IAV (22). Intriguingly, in the context of IFITM-insensitive arenavirus infection, ZMPSTE24 modulates intracellular trafficking of IFITM proteins to an early endosomal localization that increases the susceptibility of IFITM-resistance viruses (22). In summary, the anti-viral effects of ZMPSTE24 and IFITM are cumulative as well as independent, they play a crucial role in host antiviral defenses against various enveloped viruses through collaborative or independent manners. ZMPSTE24 prevents viral entry by rigidifying the cellular membrane. Further studies are required to examine whether ZMPSTE24 affects membrane composition, or if the loss of ZMPSTE24 prevents membrane stiffening by the IFITM proteins. These investigations could provide insights to explain the interdependence of the IFITMs and ZMPSTE24 across different viruses.

### 2.3 Cholesterol 25-hydroxylase

CH25H is a conserved ISG, which encodes an enzyme that synthesizes the oxysterol 25-hydroxycholesterol (25HC) from cholesterol. CH25H has been shown to have broad antiviral activity against enveloped viral infections, including VSV, EBOV, HIV-1, ZIKV, Rift Valley fever virus (RVFV), herpes simplex virus (HSV), Nipah virus, and SARS-CoV-2, by disrupting the membrane fusion process (23–25). CH25H catalyzes the conversion of cholesterol to 25-hydroxycholesterol (25HC) in the endoplasmic reticulum. 25HC inhibits sterol regulatory element-binding protein and activates acyl-CoA cholesterol acyltransferase, thereby interfering with cholesterol synthesis and uptake. This process reduces cholesterol content in cell membranes and endosomal vesicles, altering membrane dynamics (e.g., rigidity and curvature) and thus hindering viral fusion with the host cell membrane. Additionally, CH25H can directly bind to viral nonstructural proteins in an enzyme-independent manner to inhibit replication, as observed with HCV (26, 27). Recent studies have expanded its antiviral activity to include non-enveloped viruses such as reovirus (24). Furthermore, 25HC has been implicated in adaptive immune responses. For example, the accumulation of 25HC in macrophages can redirect cytotoxic CD8^+^ T cells to inhibit trogocytosis in tumors. Infections with IAV and SARS-CoV-2 cause 25HC upregulation in macrophages, suggesting that it participates in adaptive immune responses during viral infections (28, 29). *In vivo* and *in vitro* experiments have demonstrated that 25HC possesses exceptionally broad-spectrum antiviral activity, making its clinical translation research an antiviral drug of great significance. Additionally, the elevated levels of 25HC in serum during viral infection suggest its potential as a clinical biomarker during various viral infections.

### 2.4 Lymphocyte antigen 6 family member E

LY6E, a glycosylphosphatidylinositol-anchored protein, is classified as an ISG. LY6E and CH25H were identified as HRFs through the same screen based on expressing of 288 individual ISGs against VSV infection. Compared with CH25H, LY6E is less restrictive to VSV (30). LY6E specifically interferes with membrane fusion, a crucial step in the entry of enveloped viruses. It demonstrates substantial antiviral activity against all coronaviruses by impairing Spike-mediated membrane fusion through changes to host cell membrane properties, syncytia formation, and host immune responses. Thus, it effectively blocks a critical early step of viral infection (31, 32). Additionally, LY6E restricts other viruses including VSV, dengue virus (DENV), ZIKV, and HIV-1 (32, 33). This broad-spectrum activity highlights the role of LY6E as a general antiviral defense mechanism, particularly against enveloped viruses, via modulation of membrane lipid characteristics.

Conversely, some studies have shown that LY6E has pro-viral effects (e.g., for HIV-1, yellow fever virus [YFV], ZENV, and IAV) (34). For example, in the early stages of HIV-1 replication, LY6E can downregulate CD14 levels and suppress subsequent inflammatory responses, interact with syncytin-A receptors to inhibit their modulation of membrane fusion, or enhance viral Env protein localization in the viral fusion pore (34–36). Overall, LY6E exhibits a complex role in viral infections.

### 2.5 Nuclear receptor coactivator 7

NCOA7 belongs to the seven-member TLDc (Tre2/Bub2/Cdc16, lysin motif, domain catalytic) domain-containing protein family, known for its antioxidant properties. NCOA7 isoform 4 can be induced by type I IFN (37), this isoform mainly restricts viruses to enter cells through endocytosis, including VSV, IAV, SARS-CoV-2, and HCV, but not HIV-1 (37, 38). Mechanistic analyses have shown that IFN-inducible NCOA7 restricts viral entry, particularly membrane fusion, and subsequent viral trafficking processes, such as nuclear translocation of IAV. NCOA7 achieves this by promoting endolysosomal vesicle acidification and enhancing lysosomal protease activity. This regulation occurs through the modulation of vacuolar H^+^-ATPase formation and activity, which is critical for maintaining the acidic environment required for proper endosomal maturation and effective viral uncoating (39). Specifically, NCOA7 interacts with several proton pump subunits that are essential for endosomal acidification, likely through its TLDc domain (40). Other studies have demonstrated that NCOA7 plays a role in the binding of membrane-associated phosphoinositides, ensuring the correct localization and activity of vacuolar H^+^-ATPase (41). As a result, NCOA7 accelerates the turnover of viral particles, which reduces the potential for viruses to enter host cells (39). This suggests that NCOA7 is a broad-spectrum restriction factor against various viral infections by regulating endosomal function, which is crucial for viral entry.

### 2.6 Interferon-gamma inducible protein 30

IFI30, also known as gamma-interferon-inducible lysosomal thiol reductase (GILT) or IP30, is a soluble protein that is predominantly expressed in the lysosome of professional antigen-presenting cells, such as macrophages, dendritic cells and B lymphocytes. It serves as a multifunctional host protein with pivotal roles in both adaptive and innate immunities. Recent evidence has shown that IFI30 exerts antiviral activity on the entry of diverse viruses, including Lassa fever virus, EBOV, and SARS-CoV (42). These viruses enter cells by transporting to NPC1-positive lysosomes to fulfill entry into host cells via endocytosis. In contrast, viruses such as IAV, VSV and MERS-CoV can evade the antiviral effects of IFI30, as their entry occurs through cell surface, early endosome and late lysosome (42). Therefore, IFI30 inhibits the entry of specific enveloped viruses, but its broad-spectrum activity against enveloped viruses requires further investigation.

### 2.7 RAB GTPase activating protein 1 like

RABGAP1L, also known as TBC1D18 or HHL, belongs to the Tre2/Bub2/Cdc16 (TBC) domain family of proteins. It primarily regulates membrane-bound small GTPase proteins, termed RAB proteins. A recent study showed that RABGAP1L can inhibit the endocytosis of viruses such as IAV, human coronavirus (HCoV)-229E, and VSV. RABGAP1L disrupts endosome function early in the viral life cycle, specifically after attachment but before membrane fusion (43). It also acts as an ISG to enhance the immune response. However, its antiviral function does not extend to SARS-CoV-2 (43). RABGAP1L contains three structural domains: a phosphotyrosine-binding (PTB) domain, a TBC domain, and a kinase-like domain. The activated TBC domain is crucial for the viral restriction capabilities of RABGAP1L, its catalytic efficiency is closely linked to its antiviral properties (43). Another protein from the same family, TBC1D5, was identified through siRNA screens as a regulator of Rab7-mediated lysosomal degradation. TBC1D5 restricts IAV by binding to the M2 protein and promoting its lysosomal degradation (44).

## 3 HRFs that inhibit viral gene transcription and translation

After host cell entry, viruses undergo biosynthesis to produce the nucleic acids and proteins required to assemble new viral progeny. Viruses exploit the host’s cellular machinery to synthesize viral components, taking resources such as nucleotides, nucleosomes, amino acids, enzymes, and energy. To defend against this viral exploitation, host cells utilize various HRFs that specifically target viral transcription, including DEAD-box helicase 21 (DDX21), death domain-associated protein (DAXX), the structural maintenance of chromosome (SMC)5/6 complex, the transcription activation suppressor (TASOR) subunit of the human silencing hub (HUSH) complex, zinc-finger antiviral protein (ZAP) and its cofactors, APOBEC3 family proteins, and shiftless antiviral inhibitor of ribosomal frameshifting (SHFL) (**Figures 1**, **2**). In addition to their roles in regulating viral transcription, these HRFs may exert other restriction effects on different viruses. For example, DDX21 can suppress foot-and-mouth disease virus (FMDV) transcription, IAV assembly, and human cytomegalovirus (HCMV) late gene transcription.

### 3.1 DEAD-box family proteins

DDXs constitute a family of RNA helicases that belong to helicase superfamily 2. These proteins are involved in various aspects of RNA metabolism, including transcription, mRNA splicing, translation, and ribosome biogenesis. There are approximately 60 DDXs in mammalian cells. In recent decades, most DDXs have demonstrated unique and overlapping roles in regulating the innate immune response to viral infection. DDX58 (i.e., retinoic acid-inducible gene I [RIG-I]) is particularly well-known for its crucial role in recognizing viral double-stranded RNA (dsRNA) and mediating the antiviral innate immune response (45). Other DDX proteins also have key inhibitory effects on viral replication.

DDX21 restricts the infection and replication processes of various viruses, including HCMV, IAV, and SARS-CoV-2 (46–48). DDX21 binds to HCMV RNA, reducing the accumulation of R-loops and interfering with viral late gene transcription, thereby restricting viral growth (46). Upon IAV infection, DDX21 binds to IAV PB1, inhibiting polymerase assembly and reducing viral RNA levels, leading to the suppression of viral protein synthesis. It also directly interacts with and disrupts the functions of components within viral replication complexes, inhibiting replication of the viral genome. Similar to DDX1 and DDX23, DDX21 acts as a trans-acting factor in the context of the internal ribosome entry site (IRES). In particular, it inhibits the translation and replication of IRES-dependent viruses, such as FMDV, by interacting with IRES domains 2, 3, and 4 (49). Through its helicase activity, DDX17 directly interferes with viral pre-genomic RNA (pgRNA) in the cytoplasm, disrupting RNA structures essential for replication (50, 51). However, the DDX family also includes negative regulators of IFN, such as DDX19, DDX39A, DDX46, DDX5, DDX24, and DDX25. Therefore, DDX proteins play a dual role in virus-host interactions, acting as both antiviral and pro-viral factors.

### 3.2 Death domain-associated protein

DAXX is a nuclear protein closely associated with promyelocytic leukemia protein nuclear bodies (PML-NBs). It usually forms complexes with alpha-thalassemia/mental retardation X-linked (ATRX) protein, regulating diverse cellular functions such as chromatin stability, chromatin remodeling, DNA repair, cell death, and antiviral defense. Previous studies have shown that DAXX can restrict the replication of DNA viruses in the nucleus, such as human adenovirus type 5 (HAdV5) and HPV (52, 53). It can also suppress retrovirus HIV-1 in the cytoplasm through a post-translational modification called small ubiquitin-like modifier (SUMO)ylation, which is similar to ubiquitinoylation by forming covalent bonds with other proteins (on lysine) without mediating protein degradation (54). In detail, DAXX recruited TNPO3, TRIM5α, TRIM34, and possibly other proteins onto the incoming HIV-1 cores by its two SOMO-interacting motifs (SIMs) at the C-terminus, which interacted with cyclophilin A and viral capsid and increased their stability, thus preventing the uncoating and reverse transcription of HIV-1 (54). The SIMs within DAXX determine its SUMOylation-dependent transcription regulation and its subnuclear compartmentalization (55).

A recent study found that DAXX also restricts SARS-CoV-2 through a mechanism independent of the SUMOylation pathway but dependent on its Asp/Glu (D/E) domain (amino acids 414 to 505) (56). This domain is crucial for DAXX as a protein chaperone, enabling it to solubilize protein aggregates and unfold misfolded proteins. Moreover, to participate in SARS-CoV-2 replication that occurs in the cytoplasm, DAXX was translocated from the nucleus to the cytoplasm and co-expressed with dsRNA at 6 h post-infection (56). Notably, the antiviral effect of DAXX is independent of IFN signaling and stronger than that of LY6E. In response, SARS-CoV-2 has evolved a countermeasure: its papain-like protease mediates the degradation of DAXX (56). Panpain-like protease is an essential coronavirus enzyme that can process viral polyproteins to generate the viral non-structural proteins and enable viral spread. It can also cleave post-translational modification of host proteins as a means of evading host antiviral immune responses. Additionally, it can cleave ISG15 from interferon regulatory factor (IRF3), impairing the production of interferon (57, 58).

### 3.3 Structural maintenance of chromosomes 5/6 complex

SMC complex contains SMC5/6 complex, adhesion protein (cohesin, SMC1/3), and coagulation protein (condensing, SMC3/4). They are ring-shaped ATPases that play crucial roles in maintaining genome stability and regulating chromatin structure by topologically binding to chromosomes (59). The SMC5/6 complex is composed of SMC5, SMC6, and non-SMC elements 1-4 (NSE1-4). This complex is involved in DNA homologous recombination repair. Additionally, the SMC5/6 complex has been identified as a host restriction factor for several viruses, including HBV, HIV-1, HPV, and HSV-1 (60–65). The SMC5/6 complex selectively inhibits the transcription of extrachromosomal reporter genes and the HBV viral genome. Conversely, the HBV viral protein HBx recruits and induces degradation of the SMC5/6 complex via binding to DDB1, thereby counteracting its antiviral effects (60, 66). Notably, the transcriptional silencing of extrachromosomal viral DNA by SMC5/6 is unique and involves three steps. First, SMC5/6 traps viral DNA through its ATPase activity and Nse4a subunits (rather than Nse4b). Second, SMC5/6 complex localization factor 2 (SLF2) subunits (also considered human homologs of Nse6), are recruited to promyelocytic leukemia nuclear bodies. Finally, Nse2 subunits inhibit viral DNA transcription in a SUMO ligase-independent manner (66).

Researchers have elucidated how the SMC5/6 complex silences HIV-1 genes; this process involves two main pathways. First, SLF2 recruits the SMC5/6 complex to HIV-1 DNA, causing conformational compression of viral chromatin by downregulating trimethylation of histone 3 lysine 4 (H4K4me3), a marker associated with active transcription. The reduced H4K4me3 levels lead to tighter chromatin conformation, silencing the unintegrated viral genes. Second, non-structural chromatin maintenance element 2 in the SMC5/6 complex directly targets HIV-1 DNA for SUMOylation, promoting the silencing of unintegrated HIV-1 proviruses (61, 62). The HIV-1 protein Vpr and HBV protein HBx both counteract the antiviral effects of the SMC5/6 complex, facilitating viral replication (61).

### 3.4 Human silencing hub complex

The HUSH complex, composed of the TASOR, M-phase phosphoprotein 8 (MPP8 or MPHOSPH8), and periphilin, plays a critical role in transcriptional repression by recruiting the histone methyltransferase SETDB1. This recruitment leads to the deposition of the H3K9me3 modification, a marker of gene silencing, and subsequent transcriptional inhibition. The HUSH complex can inhibit the transcription of long-interspersed element-1 retrotransposon (LINE-1) and retroviruses (67). This inhibition is particularly important for the silencing of HIV proviruses, a key factor in establishing HIV latency (68). Furthermore, the HUSH complex subunit TASOR has a synergistic anti-HIV effect with the RNA adenylase called CCR4-NOT transcription complex subunit 1 (CNOT1). TASOR binds to RNA exosomes and RNA polymerase II in the extended RNA state, recruiting RNA-degrading factors. This binding inhibits gene transcription driven by the HIV long terminal repeat (LTR) promoter (69). Additionally, TRIM28, in coordination with FAM208A (another HUSH complex component), blocks HIV from exiting latency by inhibiting young retrotransposons (70). This interaction highlights the broader role of the HUSH complex in regulating HIV latency and gene silencing.

### 3.5 Zinc-finger antiviral protein

ZAP, also known as poly (adenosine diphosphate-ribose) polymerase 13 (PARP13), is an RNA-binding protein that selectively regulates the stability and translation of mRNA. ZAP is expressed as two major isoforms called ZAP-L and ZAP-S, which are produced from the same gene via alternative splicing. The two isoforms contain an N-terminal RNA binding domain with four zinc fingers.

ZAP is a broad-spectrum antiviral protein capable of resisting the replication of a variety of RNA and DNA viruses, including HIV-1, HBV, HCMV, and EBOV. It exerts antiviral defense by targeting both positive and negative single-stranded viral RNAs especially CPG-rich or cytosine-rich RNA sequences through the N-terminal domain. In the case of HIV-1, ZAP selectively binds CpG dinucleotides through its N-terminal RNA-binding domain. This binding is subjected to inhibit viral replication by inducing RNA degradation (71). For HCMV, ZAP directly bind to the UL4–UL6 transcriptional sites, thereby inhibiting viral transcription (72).

Evidence suggests that ZAP is redirected to stress granules (SGs), which provide a favorable environment for its antiviral activity. SGs were membrane-less cell compartments formed by liquid-liquid phase separation of various RNA-binding proteins (RBPs) under several stimuli. SGs participated in host antiviral immune responses by activating IFN production (73, 74). Structurally, ZAP contains a central zinc finger domain and two WWE domains. These domains, particularly the second WWE domain, bind to poly (adenosine diphosphate-ribose), promoting the relocation of ZAP to SGs. Within these granules, ZAP efficiently targets and degrades viral RNA (75). Sindbis virus (SINV) infection leads to ZAP accumulation in SGs; this accumulation plays a crucial role in determining viral viability (76).

Other factors including Riplet, TRIM25, KHNYN and Matrin 3 constitute enhancers of ZAP-mediated antiviral effects (50, 77–79). Specifically, Riplet, a key protein in activating RIG-I, was found to bind ZAP and enhance its antiviral effects against HIV-1 (77). TRIM25 inhibits EBOV through two mechanisms: targeting viral RNP for degradation (canonical, dependent on its E3 ligase activity) or modulating viral sensitivity to IFN signals (non-canonical). TRIM25-mediated antiviral activities are dependent on ZAP (80). KHNYN, an unknown protein, can bind to ZAP and promote its restriction role against HIV-1 contains clustered CpG dinucleotides, together with TRIM25 (78). Matrin 3, a nuclear matrix protein, was also reported to aid ZAP-mediated HIV restriction by expanding its targeting spectrum from viral unspliced RNAs to multiply-spliced RNAs (79). The exact relations between these ZAP cofactors are not clear. ZAP’s central role in cellular antiviral programs has led to recent investigations regarding the feasibility of utilizing its characteristics to develop attenuated RNA vaccines (81).

### 3.6 Apolipoprotein B mRNA-editing enzyme catalytic polypeptide 3

The APOBEC3 proteins are a family of deoxycytidine deaminases comprising of seven members A3A, A3B, A3C, A3D, A3F, A3G, and A3H. These proteins inhibit a broad range of DNA viruses, RNA viruses, and retroviruses through both deaminase-dependent or -independent mechanisms (82). Among them, A3D, A3F, A3G and A3H are particularly effective as retrovirus restriction factors. They restrict viral replication by introducing cytosine-to-uracil hypermutations in viral complementary DNA, resulting in aberrant viral intermediates and impaired reverse transcription, a process reliant on their deaminase activity (83).

Specifically, A3G binds HIV-1 and disrupts its replication, while A3A lacks activity against retroviruses (84). However, A3A is a potent inhibitor of parvovirus, it inhibits parvovirus DNA replication in a deaminase-independent manner (85). Previous studies found that A3G prefers recognizing ssDNA and RNA with stem-loop structures. It restricts HIV infection primarily by directly binding to viral RNA or reverse transcriptase, thereby interfering with HIV-1 DNA synthesis in a manner distinct from its deaminase activity (83). Additionally, A3G inhibits HIV-1 integration by deaminating the 3’ LTR, which increases integration site diversity (86). APOBEC3 proteins can also be packaged into virions through interactions with various RNA (viral RNA, cellular mRNA, or small noncoding RNA) in a nucleocapsid-dependent manner. Furthermore, A3G inhibits HBV infection by binding to approximately 35% of HBV genome and preferentially deaminating the third cytosine in the 5’ CCC of viral DNA (87). A3G also directly inhibits HBV S gene promotor activity or impedes the interaction between its positive regulator, heterogeneous nuclear ribonucleoprotein K, and enhancer II. Moreover, A3G has been shown to inhibit the replication of enterovirus 68 (EV-68) by competitively binding to the 5’ untranslated region (UTR) with host factor poly(C)-binding protein 1 (PCBP1) (88).

To counteract the antiviral activities of APOBEC3 proteins, the HIV viral infectivity factor (Vif) induces their degradation through a mechanism dependent on the ubiquitin-proteasome pathway (89). By targeting Vif-A3G interaction, antivirals against HIV like IMB-26, IMB-35, RN-18, and RN-19 have been identified (10, 90–92). Further studies are required to identify potential therapeutics of HBV or EV-68 by targeting host-viral interaction.

### 3.7 Shiftless antiviral inhibitor of ribosomal frameshifting

SHFL, also known as C19orf66, RyDEN, IRAV, or FLJ11286, is an ISG recognized for its broad-spectrum antiviral activity. SHFL inhibits a wide range of viruses, including DENV, HIV-1, Kaposi’s sarcoma-associated herpesvirus (KSHV), HCV, ZIKV, Japanese encephalitis virus (JEV), and YFV, through diverse mechanisms (93). Studies in mouse models have demonstrated that SHFL has a neuroprotective role during ZIKV infection (94). Specifically, SHFL is the first identified host factor to target -1 programmed ribosomal frameshifting (-1PRF), inhibiting the translational recoding in multiple viruses (93, 95). In addition to this unique mechanism, SHFL restricts viral gene transcription and promotes viral genome degradation by interacting with viral proteins. For example, SHFL induces the K48-ubiquitin protease-mediated degradation of the 3D protein of EV-71. It degrades the NS3 and NS5A proteins of ZIKV. SHFL targets the open reading frame (ORF)50 and ORF70 proteins of KSHV, and binds to the NS3 protein of JEV (95–97). Moreover, SHFL interacts with the viral RNA of *Flaviviridae*, inhibiting viral genome replication (94). Thus, the multifunction antiviral activity of SHFL depends on the specific viral component it targets.

In other contexts, SHFL restricts viral RNA or protein stability and gene expression by binding to host cellular pathway regulators. These include ubiquitin ligases, ATPases, RNA-binding proteins, and glycolysis-related proteins, to modulate these cellular activities during viral infection (98).

## 4 HRFs that inhibit viral assembly and release

Following biosynthesis, newly synthesized viral proteins and genetic material are assembled into structurally intact and infectious progeny viruses within the host cell. This assembly process can vary depending on the type of virus-RNA viruses and DNA viruses have distinct mechanisms and locations for assembly. Once assembly, the progeny viruses are released from the host cell to propagate further infection.

### 4.1 Bone marrow stromal cell antigen 2

BST-2, also known as tetherin, CD317, or HM1.24, is a type II transmembrane glycoprotein with two membrane-associated domains: an intracellular N-terminal (NT) domain and an extracellular coiled-coil (CC) domain. The NT domain facilitates BST-2 cycling between plasma and endosomal membranes, while the CC domain contributes to its structural conformation and dimerization.

BST-2 is an ISG induced primarily by type I IFN. It exerts broad-spectrum antiviral activity by inhibiting the release of various viruses, including alphavirus, HIV, IAV, DENV, EBOV, RSV, HBV, HCV, chikungunya virus (CHIKV), and SARS-CoV-2. BST-2 inhibits viral replication primarily through tethering virions. BST-2 uses its N-terminal transmembrane domain and C-terminal glycosylphosphatidylinositol anchor to tether progeny virus particles to the cell surface. This retention prevents the release of newly assembled infectious virions, effectively halting viral propagation (99, 100). The anchored virions are subsequently sequestered in tetherin-positive compartments, further restricting viral spread (101). Human BST-2 exists in two isoforms: long (L-tetherin) and short (S-tetherin), both of which can inhibit viral virions release. L-tetherin contains a serine-threonine-serine motif, essential for endocytic recycling and virus-induced NF-κB activation (102). L-tetherin exhibits stronger antiviral activity against alphaviruses, attributed to this motif. S-tetherin lacks the first 12 amino acids of the cytoplastic tail of L-tetherin, including the tyrosine motif leading to a reduced sensitivity to HIV-1’s Vpu protein. This reduced sensitivity emerged during zoonotic transmission of HIV from chimpanzees (102).

The antiviral activity of BST-2 was first reported in HIV, and it has now been found to inhibit HIV infection through several mechanisms. First, it directly interacts with both nascent and mature HIV particles, tethering them to the host cell membrane and preventing their release, which is particularly effective against enveloped viruses. Second, it promotes the formation of virus-containing compartments (VCCs) in HIV-infected macrophages, sequestering viral particles and restricting their spread to neighboring cells (103). Third, increased methylation of BST-2 in HIV-infected patients downregulates its mRNA level, leading to worse clinical outcomes in HIV-1 infections. This finding highlights the importance of post-translational modifications in regulating gene expression (104). Finally, BST-2 can initiate intracellular signaling pathways and inflammatory responses to inhibit HIV-1 assembly by sensing viral signals and activating NF-kB (105).

Additionally, BST-2 plays a complex role in regulating immune responses and viral propagation, acting as a double-edged sword due to its involvement in both the activation and regulation of immune signals. BST-2 promotes pro-inflammatory signals by stimulating NF-κB activation, enhancing the immune response against infections, and amplifying IFN signals through a positive feedback loop, thereby strengthening antiviral defenses. Conversely, BST-2 helps prevent excessive immune responses—which could lead to autoimmune diseases—by mediating the degradation of mitochondrial antiviral signaling protein (MAVS) or restricting the production of LINE-1 RNA (106, 107). LINE-1, an ancient retrovirus integrated into the host genome, is capable of autonomous reproduction. BST-2 can suppress LINE-1 retro-transposition by reducing the promoter activity of its 5’ UTR (108).

High-throughput screening has identified antiviral compounds that target HIV Vpu-BST-2 interaction. For example, 2-thio-6-azauridine (NSC-146268) protects BST-2 from degradation mediated by Vpu protein (109). Other compounds, such as IMB-LA, BST2-TM-P1 (contain BST-2 transmembrane domain sequences), and Y-39983 HCl combat HIV infection by competing with Vpu for binding to BST-2 (91, 92, 110).

### 4.2 Membrane-associated RING-CH-type finger 8

The MARCH family consists of 11 members within the RING-finger E3 ligase family. These proteins typically contain a C4HC3 RING-finger domain at the N-terminal, which facilitates the removal of transmembrane proteins (such as major histocompatibility complex [MHC]-II) from the plasma membrane by mediating substrate ubiquitination. MARCH8, the first identified cellular modulator of immune recognition within the human genome, exerts broad-spectrum antiviral effects against the glycoproteins of enveloped viruses, including rabies virus, Ross River virus (RRV) CHIKV, lymphocytic choriomeningitis virus (LCMV), SARS-CoV, SARS-CoV-2, HIV-1, and EBOV (111–113). Recent studies have shown that MARCH8 inhibits the assembly and release of IAV particles. Specifically, MARCH8 mediates K63-linked polyubiquitination of the 78th lysine residue of the viral M2 protein. This modification is crucial for the targeted trafficking of M2 protein from the viral surface of the H1N1 A/WSN/33 virus to lysosomes for subsequent degradation. This process disrupts the viral life cycle by impairing viral particle assembly and disrupting viral envelope division, thus hindering the release of progeny viruses (114). However, another study showed contradictory findings, whereby MARCH8 functions through its N-terminal cytoplasmic domain, rather than targeting virus-coated glycoproteins such as hemagglutinin (HA), neuraminidase protein (NA), matrix protein 1 (M1), and matrix protein 2 (M2) (115).

### 4.3 Sterile alpha motif and histidine-aspartic acid domain-containing protein 1

SAMHD1 is a ubiquitously expressed enzyme with adenosine triphosphate (ATP) hydrolase activity, regulates intracellular dephosphorylation of deoxynucleotide triphosphates (dNTPs). SAMHD1 was initially identified as a host restriction factor because it can effectively inhibit HIV-1 replication in myeloid cells and resting CD4+ T cells. It restricts retroviruses by reducing cellular dNTP concentrations, which are essential for efficient viral reverse transcription. Additionally, SAMHD1 exerts antiviral effects through several mechanisms independent of its dNTPase activity. For instance, SAMHD1 restricts the replication of lipid-dependent viruses, such as flaviviruses and HCV, by interfering with lipid biosynthesis pathways via its C-terminal (116). Specifically, SAMHD1 down-regulates sterol regulatory element binding protein (SREBP1), a key regulator of cholesterol production and low-density lipoprotein (LDL) intake, thereby inhibiting lipid droplet formation and reducing viral infectivity (116). Moreover, SAMHD1 undergoes SUMOylation, a post-translational modification involving the attachment of small ubiquitin-like modifier proteins. SUMOylation at lysine 595, mediated by the SIM2 motif, enhances SAMHD’s ability to restrict HIV-1 (117). Conversely, phosphorylation of SAMHD1 at threonine 592 reduced its antiviral potent (118).

SAMHD1 also inhibits various EVs, including EV-68, EV-71, and CA6. A recent study demonstrated that SAMHD1 robustly inhibits EV71 assembly by binding to the VP1 protein, competing with the VP1–VP2 interaction required to form infectious viral particles (119). Targeting SAMHD1-VP1 interaction may lead to potential therapeutics for EV71.

Together, SAMHD1 acts as a versatile antiviral protein by targeting multiple stages of the viral life cycle, including dNTP depletion, inhibition of lipid synthesis, protein modification, and direct interference with viral protein interactions. Further studies are needed to explore potential antivirals that can enhance SAMHD1’s activity or modulate its post-translational modification, such as phosphorylation or SUMOylation, to combat a broad spectrum of viral infections.

## 5 Other HRFs

In addition to HRFs that directly target specific stages of the viral life cycle, there are other functional RNAs and proteins that disrupt viral infections indirectly. These factors do not necessarily target viral processes at a specific stage, but instead operate by modulating host cellular pathways (**Table 1**). This can include enhancing immune responses, regulating the cellular stress response, or altering the host cell’s metabolic state, among other strategies.

**Table 1:** A summary of HRFs.

Replication Cycle	Host Factor	Virus Type	Mechanism	Reference
**Invasion**	IFITM1~3	Enveloped RNA viruses including orthomyxoviruses, flaviviruses, rhabdoviruses, bunyaviruses, filoviruses, alphaviruses, coronaviruses, retroviruses; DNA viruses such as poxviruses, iridoviruses; non-enveloped RNA viruses like reoviruses	Inactivates viral envelope glycoproteins	(13, 15, 17, 18, 120, 121).
	ZMPSTE24	Enveloped viruses including orthomyxoviruses (IAV), coronaviruses (SARS-CoV-2), flaviviruses (EBOV, ZIKV), alphaviruses (SINV), vesculoviruses (VSV), poxviruses (cowpox, vaccinia), arenaviruses	Co-operates with IFITM3	(20–22)
	CH25H	Enveloped and non-enveloped viruses	Prevents cholesterol synthesis	(23–26, 28, 29)
	LY6E	Vesculoviruses (VSV), flaviviruses, retroviruses (HIV-1), coronaviruses (HCoV, SARS-CoV-2)	Adjusting membrane lipid properties	(30–33)
	NCOA7	Orthomyxoviruses (IAV), Hepadnaviruses (HCV), vesculoviruses (VSV), coronaviruses (SARS-CoV-2)	Interacts with V-ATPase, and degrades viral protein	(37–39)
	GLIT/IFI30	Flaviviruses (EBOV), picornaviruses (LASV), retroviruses (HIV-1), coronaviruses (SARS-CoV-2)	Degrades cathepsin-L, restricting lysosomes-mediated viral entry	(42)
	RABGAP1L	Orthomyxoviruses (IAV), coronaviruses (SARS-CoV-2)	Reduces viral endocytosis and transportation	(43, 44)
	DDX17	Hepadnaviruses (HBV)	Directly interferes pgRNA	(51)
	DDX21	Picornaviruses (EVs, FMDV), orthomyxoviruses (IAV), coronaviruses (SARS-CoV-2)	Inhibits IRES-dependent viral replication and transcription, targeting viral protein	(49)
	DAXX	coronaviruses (SARS-CoV-2, SARS-CoV), adenoviruses (HAdV-5), herpesviruses (HPV), retroviruses (HIV-1)	Depends on its D/E domains and molecular chaperone activities	(52–54, 56)
	SMC5/6 Complex	Retroviruses (HIV-1), hepadnaviruses (HBV), herpesviruses (HPV, HSV)	Silence viral genes by SUMOylation or NSE2-dependent pathways; or capture viral DNA outside the chromosomes	(62, 64, 66)
	TASOR	Retroviruses (HIV-1)	Recruiting RNA degrading proteins together with CNOT1	(69)
	ZAP	Herpesviruses (HCMV, MHV-68); hepadnaviruses (HBV, HEV); poxviruses (MVA); parvoviruses (MVM); filoviruses (EBOV, MARV); orthomyxoviruses (IAV); coronaviruses (SARS-CoV-2); togaviruses; retroviruses; flaviviruses; picornaviruses (CVB3, EV-A71)	Target viral UL4-UL6 transcripts	(72, 122)
	A3G	Retroviruses (HIV-1), hepadnaviruses (HBV), picornaviruses (EV-68)	Catalyze deamination of vDNA; bind viral RNA, enzymes, or compete pro-viral PCBP1	(83, 86–88)
	SHFL	Retroviruses (HIV-1), picornaviruses (EV-71), flaviviruses (ZIKV, JEV), herpesviruses (KSHV)	Target viral protein for degradation; or bind vRNA	(94–97, 123)
**Assembly**	SAMHD1	Picornaviruses (EVs)	Bind to VP1 and compete for VP1-VP2 interaction	(119)
**Release**	BST2/tetherin	Retroviruses, arenaviruses (Lassa and Machupo), herpesviruses (KSHV), rhabdoviruses (VSV), paramyxoviruses (SeV, Nipah), orthomyxoviruses (IAV), flaviviruses (HCV), arenaviruses (Lassa and Machupo)	Anchored to cell membrane surface	(107, 124)
	MARCH8	Orthomyxoviruses (IAV), coronaviruses (SARS-CoV-2), retroviruses (HIV-1)	Catalyzes K63-linked polyubiquitination of viral proteins; blocks viral protein	(112–114)
	TRIM7	Picornaviruses (EVs, EMCV), coronaviruses (SARS-CoV-2)	Targets viral protein for degradation or triggers innate IFN-β pathway	(125–127)
	TRIM14	orthomyxoviruses (IAV), flaviviruses (EBOV, HCV), herpesviruses (HSV-1), hepadnaviruses (HBV), alphaviruses (SINV)	Enhance IFN responses; or target viral proteins for degradation	(128–135)
	TRIM25	Paramyxoviruses (SeV), orthomyxoviruses (IAV), flaviviruses (EBOV), coronaviruses (SARS-CoV-2)	Enhance IFN responses; as RBPs; or target viral proteins for degradation	(80, 136–139)
	TDRD3 G3BP1	Picornaviruses (EVs), SeV, rhabdoviruses (VSV)	Induces SGs and activates IFN responses	(140, 141)
	OPTN	Herpesviruses (HSV-1)	Target viral proteins for autophagy and degradation	(142)
	FAM111A	polyomaviruses (SV40 polyomavirus), poxviruses (positive poxvirus), flaviviruses (ZIKV)	Activates RFC3 to inhibit viral replication	(143) (143–147).

### 5.1 IFN-associated HRFs

#### 5.1.1 Tripartite motif-containing proteins

The TRIM (also known as the RING, B-box, coiled-coil [RBCC] motif) family is a group of proteins with E3 ubiquitin ligase activity, characterized by the presence of a RING domain, B-Box, and coiled-coil domains. These proteins regulate various physiological functions within cells and play key roles in innate immunity, inflammation, and anti-infection responses. TRIM proteins exert their antiviral effects primarily through their E3 ligase activities, but they utilize several mechanisms due to the diversity of their substrates. These mechanisms include: 1) ubiquitination and degradation of viral proteins, and 2) activation of immune signaling pathways. Some TRIMs can also bind viral RNA and function as RNA-binding proteins (RBPs). Several examples of TRIMs closely involved in antiviral immune responses are discussed below.

TRIM7, also known as glycogenin-interacting protein (GNIP), exists in four isoforms: GNIP1-3 and a short form (148). By catalyzing the ubiquitination of various proteins (e.g., MAVS, STING, NF-κB, and IRF3), TRIM7 activates the IFN-β response and Toll-like receptor (TLR)4 signaling (125). Conversely, the short form of TRIM7 can downregulate the NF-κB pathway, RIG-I/MAVS, and cGAS-STING signaling (149–151). In addition to the modulation of cell-intrinsic immune pathways, TRIM7 targets proteins expressed by numerous viruses. For example, TRIM7 interacts with 2BC protein of enteroviruses through its C-terminal PRYSPRY domain, while its RING domain mediates K48 ubiquitination of 2BC (126). This ubiquitination induces 2BC degradation through the proteasome, thereby impairing the membrane remodeling process essential for viral replication (126). The evidence above confirms that TRIM7 is a key antiviral host factor involved in controlling EVs.

TRIM14 is an HRF with a broad antiviral spectrum, inhibiting IAV, EBOV, HSV-1, HBV, HCV, and SINV through multiple mechanisms (128–133). As a STAT1-dependent ISG, it commonly exerts antiviral effects by triggering innate immune defense systems and inflammatory responses. It localizes to the outer membrane of mitochondria and interacts with MAVS, leading to MAVS signalosome and the activation of IRF3 and NF-κB (134). Recent studies have revealed the role of TRIM14 in linking autophagy and IFN production by stabilizing cGAS against p62-induced degradation. This is achieved through TRIM14’s recruitment of USP14, which cleaves the ubiquitin chain at site K414 on cGAS (131). Moreover, TRIM14 directly binds to viral proteins, such as the nucleoprotein of EBOV (130). TRIM14 also targets the HBV HBx protein and HCV NS5A protein for degradation through its SPRY domain. These interactions restrict HBV replication by impairing the formation of the SMC-HBx-DDB1 complex and inhibiting HCV infection through mechanisms independent of IFN signals (129, 132, 133).

TRIM25 is a multifaceted protein with significant antiviral effects across various mechanisms. First, it is a key regulator of innate immune responses, enhancing RIG-I/MAVS signaling by inducing K63-linked ubiquitination of RIG-I. This activity is medicated through the interaction of TRIM25’s SPRY domain with the caspase recruitment (CARD) domain of RIG-I (136). This interaction enhances RIG-I/MAVS-mediated IFN production (136). Second, TRIM25 has been shown to restrict RNA viruses, such as SeV, through co-condensing with Ras-GTPase-activating protein SH3-domain-binding protein 1 (G3BP1) in SGs upon stimulation with dsRNA such as poly(I:C). Third, TRIM25 directly targets viral RNA (as an RBP) or proteins for degradation. For example, TRIM25 binds and destabilizes IAV mRNAs without disrupting transcription (137). Another high-throughput study revealed TRIM25 as an RBP that binds to SARS-CoV-2 RNAs. Notably, by cooperating with ZAP, TRIM25 can restrict the EBOV ribonucleoprotein complex and enhance type I IFN signals; its activity is regulated by the CpG dinucleotide content of the viral genome (80). It also inhibits HBV replication by binding to and mediating the degradation of HBx and promoting pgRNA recognition by RIG-I (138).

In summary, most TRIM proteins represent a highly diverse and crucial component of the host’s antiviral defense system. These proteins exhibit a broad range of antiviral mechanisms that can operate both dependently and independently of triggering innate immune responses.

#### 5.1.2 The family with sequence similarity 111 trypsin-like peptidase A

FAM111A is a tryptase-like serine protease located in the nucleus. It constitutes an HRF with broad-spectrum antiviral effects against viruses such as SV40 polyomavirus, poxviruses, and ZIKV (143–147). However, its antiviral mechanism remains unclear. Recent studies have shown that upon infection with ZIKV, FAM111A is activated by IRF2, which enhances replication factor C subunit 3 (RFC3) signaling and inhibits viral replication (143). Additionally, FAM111A overactivation can lead to hydrolysis of the nuclear pore complex (NPC) factors germinal center-associated nuclear protein (GANP) and nucleoporin protein 62 (NUP62) by its protease activity. This hydrolysis impairs nuclear barrier function, perturbs cell cycle entry into the S phase, decreases cell motility, and ultimately affects nuclear permeability; thus, it inhibits the replication of SV40 HR mutants (152).

Furthermore, several previously mentioned HRFs are also involved in IFN responses, including G3BP1, MARCH8, OASL-IT1, TRIM7, DDX50, and DDX60 (140, 153–156). These HRFs contribute to antiviral immunity by modulating IFN production and enhancing ISGs expression, which plays a critical role in limiting viral replication. In addition, in silico screening of compounds that bind to IFNAR2 binding pocket has identified potential antiviral agents effective against HBV and HCV. Similar approaches could help identify additional IFN-related or ISG-related HTAs, offering promising avenues for the development of novel antiviral therapies (157).

### 5.2 SG formation-related HRFs

Tudor domain-containing protein 3 (TDRD3) is a methyl-reading protein characterized by a Tudor domain, a putative nucleic acid recognition motif, and a ubiquitin-related domain. TDRD3 plays an important antiviral role by regulating the formation of SGs. Another key protein involved in SG formation, G3BP1, undergoes methylation at arginine residues, which influences the kinetics of SG formation. Studies have shown that TDRD3 and G3BP1 co-aggregate in SGs during EVs infection. This co-aggregation enhances the antiviral response by activating various IFN signaling pathways, which are crucial for inhibiting EV replication (141). Conversely, EVs cleave TDRD3 and G3BP1 via their 2A and 3C proteases. These cleavages inhibit SG formation, undermining the host cell’s antiviral defenses (141, 158). Additionally, G3BP1 acts as a positive regulator of RIG-I, affecting the replication of RNA viruses such as SeV and VSV (140). TDRD3 and G3BP1 play pivotal roles in antiviral defense through their involvement in SG dynamics and IFN signaling pathways. Their co-aggregation in response to viral infection and subsequent activation of distinct IFN pathways highlight their importance in inhibiting viral replication. However, EVs counteract these defenses by cleaving TDRD3 and G3BP1, illustrating the ongoing battle between host antiviral mechanisms and viral evasion strategies.

### 5.3 OPTN: an autophagy-related HRF

Optineurin (OPTN), a host autophagy adaptor protein, is involved in clearing cytosolic bacteria and viruses. As reported, OPTN has versatile functions in autophagy and mitophagy. Firstly, it can initiate autophagy at an early stage when autophagosomal membranes form. Specifically, it can be recruited and recognize ubiquitinated cargos, allowing selective autophagy to occur. It prefers to bind linear polyubiquitin chains and K63 chains rather than K48 chains or monoubiquitin-modified substrates. Thirdly, it interacts with microtubule-associated protein 1 light chain 3 (LC3)-II-conjugated-autophagic membrane via its LC3-interacting region (LIR), to link the ubiquitinated cargos onto the autophagic membranes. This provides the basis for autophagosome formation. As continuing, it recruits other key proteins like the unc-51 like autophagy activating kinase 1 (ULK1) complex to initiate phagophore biosynthesis and redirect LC3 lipidation by recruiting the ATG12–ATG5-ATG16L1 complex (159).

Recent studies have shown that OPTN mediates the autophagic and ubiquitin-dependent degradation of HSV-1 by targeting its shell protein (VP16) and fusion glycoprotein (gB) (142). gB, locates on the envelope of HSV-1 virions, is responsible for fusion with host cell membranes upon entry, while VP16 is a transactivating factor that hijacks HRFs to enhance viral gene transcription. During viral infection, viral proteins are ubiquitinated and these cargoes are recognized by autophagy receptors like OPTN. Meanwhile, TANK-binding kinase 1 (TBK1) promotes receptor affinity by phosphorylating it. Then OPTN recruits the cargos onto phagosomal membranes and initiates further phagophore formation by recruiting ULK1. This mechanism restricts viral transmission and protects the host’s central nervous system (142). In OPTN knockout mice, host immunity is impaired, and HSV-1 infection leads to severe CNS infections and death, highlighting that OPTN is a necessary HRF against neuroinvasive HSV-1 infections (142). As a countermeasure, HSV-1 encodes the virulence factor γ_1_34.5, which inhibits autophagy. Additionally, HSV-1 down-regulates OPTN phosphorylation by inhibiting TBK1, further evading host defenses. Although VP16 is involved in viral replication and gB plays a role in viral entry, the precise stage of the viral life cycle targeted by OPTN remains unclear. Further studies are needed to determine whether OPTN’s protective role extends to infection of other virus types.

## 6 Summary

Numerous HRFs limit viral infection by targeting different stages of the viral lifecycle, including viral entry, protein or nucleic acid synthesis, assembly and release. Moreover, the activation of host signaling pathways after infection can also prevent viral infection. These pathways include innate antiviral responses, post-translational modification, autophagy, transcription, and translation (**Table 1**, **Figures 1**, **2**). In summary, the host-virus interaction is a dynamic and constantly evolving process and further in-depth studies of the antiviral mechanisms of HRFs will enhance our understanding of host antiviral responses and viral countermeasures.

By targeting host-viral interaction, a series of effective antiviral drugs have been identified. However, there is still a long journey ahead, from understanding the molecular mechanism by which host factors restrict viral replication to ultimately discovering potential compounds against viruses. This review provides new insights into how the host restriction factors work, and facilitates better prevention and treatment strategies for viral infections, while also opening new avenues for host-targeting antiviral research.
